# Blending Face-to-Face and Internet-Based Interventions for the Treatment of Mental Disorders in Adults: Systematic Review

**DOI:** 10.2196/jmir.6588

**Published:** 2017-09-15

**Authors:** Doris Erbe, Hans-Christoph Eichert, Heleen Riper, David Daniel Ebert

**Affiliations:** ^1^ Department of Special Education and Rehabilitation University of Cologne Cologne Germany; ^2^ Department of Clinical Psychology Vrije Universiteit Amsterdam Amsterdam Netherlands; ^3^ Department of Clinical Psychology and Psychotherapy Friedrich-Alexander University Erlangen-Nuremberg Erlangen Germany

**Keywords:** mental health, Internet, psychotherapy, blended treatment

## Abstract

**Background:**

Many studies have provided evidence for the effectiveness of Internet-based stand-alone interventions for mental disorders. A newer form of intervention combines the strengths of face-to-face (f2f) and Internet approaches (*blended interventions*).

**Objective:**

The aim of this review was to provide an overview of (1) the different formats of blended treatments for adults, (2) the stage of treatment in which these are applied, (3) their objective in combining face-to-face and Internet-based approaches, and (4) their effectiveness.

**Methods:**

Studies on blended concepts were identified through systematic searches in the MEDLINE, PsycINFO, Cochrane, and PubMed databases. Keywords included terms indicating face-to-face interventions (“inpatient,” “outpatient,” “face-to-face,” or “residential treatment”), which were combined with terms indicating Internet treatment (“internet,” “online,” or “web”) and terms indicating mental disorders (“mental health,” “depression,” “anxiety,” or “substance abuse”). We focused on three of the most common mental disorders (depression, anxiety, and substance abuse).

**Results:**

We identified 64 publications describing 44 studies, 27 of which were randomized controlled trials (RCTs). Results suggest that, compared with stand-alone face-to-face therapy, blended therapy may save clinician time, lead to lower dropout rates and greater abstinence rates of patients with substance abuse, or help maintain initially achieved changes within psychotherapy in the long-term effects of inpatient therapy. However, there is a lack of comparative outcome studies investigating the superiority of the outcomes of blended treatments in comparison with classic face-to-face or Internet-based treatments, as well as of studies identifying the optimal ratio of face-to-face and Internet sessions.

**Conclusions:**

Several studies have shown that, for common mental health disorders, blended interventions are feasible and can be more effective compared with no treatment controls. However, more RCTs on effectiveness and cost-effectiveness of blended treatments, especially compared with nonblended treatments are necessary.

## Introduction

### Background

Empirical evidence suggests that Internet-based psychological interventions can be used to effectively treat adults, adolescents, and children for various mental disorders such as depression, anxiety, or problematic substance use [1-6]. Such interventions have several advantages over conventional face-to-face (f2f) interventions. For example, Internet-based interventions can be administered over long distances, may save therapists’ time, allow both patients and clinicians to work at their own pace, save traveling time, and reduce the stigma of having a mental disorder or going to a psychologist or therapist [7,8].

On the other hand, Internet-based interventions may also have disadvantages when compared with face-to-face therapies. For instance, Internet interventions may require certain abilities such as computer and Internet skills, reading and writing skills, and, in comparison with traditional therapy settings, more self-reflection and eloquence when talking about one’s thoughts and feelings. Furthermore, it has been argued that this type of intervention may make it difficult for therapists to adequately react to crisis situations such as suicidality because nonverbal cues are missing as additional information when assessing whether dissociation of suicidal thoughts is possible [9]. Also, negative effects such as frustration due to failure or time pressure might be associated with the Internet treatment format [10-13]. These disadvantages often lead to stand-alone Internet treatments being regarded as low-threshold interventions for milder cases of mental disorders, whereas face-to-face therapy and pharmacotherapy are often regarded as options of choice for more severe symptoms [14]. In addition to guided or unguided Internet treatment as a stand-alone intervention, a newer treatment approach combines face-to-face sessions with Internet-based sessions into one integrated treatment. This approach is usually called *blended treatment* and aims at retaining the positive aspects associated with both forms of therapy while mitigating the disadvantages [15-17]. There are different potential advantages of blending Internet and face-to-face treatments. For example, viewed from a cost-effectiveness perspective, blended treatments could possibly diminish the number of face-to-face contacts and thereby decrease the overall costs of treatment. Blended treatments could also increase the effectiveness by, for example, increasing frequency from one to two sessions per week [18] through adding Internet sessions to face-to-face interventions, thereby increasing intensity and success without additional costs. Furthermore, adding Internet interventions might improve transfer to everyday life as Internet or mobile elements could be used to support behavior change during face-to-face sessions and thereby increase effectiveness of face-to-face psychotherapy. Blended format could potentially also reach individuals for whom either purely delivered Internet-based or pure face-to-face approaches are not a suitable treatment option and thereby increase the utilization of effective treatments.

As both face-to-face and Internet-based psychotherapy have advantages and disadvantages, combining the two approaches in a blended treatment might combine the best of two worlds.

### Definition of Blended Interventions

As a clear definition of blended interventions is still missing [17], we define blended interventions in this study as treatment programs that use elements of both face-to-face and Internet-based interventions, including both the integrated and the sequential use of both treatment formats. Nonblended interventions comprise face-to-face treatments only or stand-alone Internet treatments. Within blended treatments, face-to-face contacts may be added to Internet interventions or, vice versa, the Internet-based part may be arranged as an adjunct to existing face-to-face programs. Internet interventions may also be arranged as an aftercare or maintenance element after acute phase treatment or as an early step within a stepped care program. *Stepped care* refers to a treatment program where interventions start with the least intensive and least costly treatment that is likely to work, progressing step-by-step with more intensive interventions for those patients insufficiently helped by the first or previous intervention. Blended stepped care treatments involve Internet-based treatments as one step within the sequence. See Textbox 1 for a subject index of types of blended interventions.

**Subject index**

Nonblended interventions: Face-to-face (f2f) treatments or stand-alone Internet-treatments only

Blended interventions: Treatment programs that use elements of both face-to-face and Internet-based interventions, including sequential use of both forms of treatment

- Integrated blended interventions: Blended treatments where the Internet-based intervention part is arranged as an adjunct to face-to-face programs or vice versa so that face-to-face and Internet-based elements are provided within the same period. In integrated blended interventions, the focus can be either on the face-to-face treatment or on the Internet-based intervention.
- Sequential blended interventions: Blended treatments where the Internet-based intervention part is arranged before or after the face-to-face treatment such as within stepped care approaches or aftercare interventions that directly follow the face-to-face intervention.

### Objective

This systematic review supplies an overview of research into blended interventions for mental health. Specifically, we focus on the following questions: (1) Which blended intervention concepts have been proposed in the researched literature in the treatment of common mental disorders (anxiety disorders, depression, and substance abuse)? (2) In which stage of treatment (such as first step, acute phase treatment, and maintenance phase) do the Internet interventions take place? (3) Which types of problem and target group do the blended interventions focus on? (4) What is the objective in combining face-to-face and Internet-based approaches? and (5) What evidence is there for the effectiveness of blended interventions?

## Methods

### Search Strategy

Studies of potential relevance were identified using a systematic search in the MEDLINE, PsycINFO, Cochrane, and PubMed databases. All studies up to December 2015 were included. Searches were performed using keywords indicating face-to-face interventions (“inpatient,” “outpatient,” “face to face,” or “residential treatment”), which were combined with terms indicating Internet treatment (“internet,” “online,” or “web”) and terms indicating mental disorders (“mental health,” “depression,” “anxiety,” or “substance abuse”). We focused on three of the most common mental disorders: depression, anxiety, and substance abuse. The bibliographies of the identified studies revealed additional sources.

### Study Selection

Studies were included if they met the following inclusion criteria: (1) the study was on an intervention that was based on both an Internet and a face-to-face treatment element that was either integrated or delivered sequentially, (2) the study involved treatment for adults with depression, anxiety, or substance abuse, and (3) the study was published in English or German. Studies with mere self-help interventions were excluded.

After the initial database search and removal of duplicates, the title and abstract of the remaining studies were rated for the inclusion criteria independently by the first and second author. Interrater reliability was good (kappa, κ=.825, *P*<.001). The first and second authors then used the inclusion criteria to independently review all full-text publications judged as relevant in the title and abstract screening. Consensus through discussion was sought in cases of disagreement. If no agreement could be reached through discussion, the last author made a decisive judgment. This was the case for 2 studies.

In November 2016, we started a second search in the database of PubMed, searching for studies citing the studies we had found in the initial search. The extraction strategies as of the initial search were used for those studies, including the independent rating of studies by the first and second authors in the first and second steps.

### Quality Assessment

New quality assessment criteria were created since there are no current guidelines for assessing the quality of blended intervention studies. The quality of each study was rated on five aspects: study design, randomization of study conditions, report of statistics, sample size (studies powered to detect effect sizes of a minimal important difference between blended and nonblended interventions of a priori defined Cohen *d* of 0.35 were classified as high quality), and existence of a nonblended active control group. Each aspect was rated on a 3-point scale (0-2), providing a score with a range from 0 to10 (see Table 1 for detailed descriptions of quality assessment). Studies obtaining a total score above two-thirds of the maximum score (ie, >7) were considered high quality studies.

**Table 1:** Study assessment criteria for scoring.

Aspect	Scoring
Study design	Which design did the study have? 2=controlled trial, 1=pre-post, 0=case study or unclear.
Randomization	Were participants randomized to conditions (depending on design)? 2=yes, 0=no.
Report of statistics	Are relevant statistics reported? 2=mean and standard deviation for outcome measures, effect sizes, and *P* values for significant differences are reported, 1=is lacking any of these, 0=lack several of these.
Sample size	Was the sample size adequate to detect effect sizes of *d*=0.35? 2=264 or more, 1=more than 132, 0=132 or less
Nonblended control group	Did the design involve a nonblended active control group? 2=nonblended control group with same number of sessions, 1=active control group without assured same number of sessions, 0=no active control group

## Results

### Study selection

**Figure 1:**
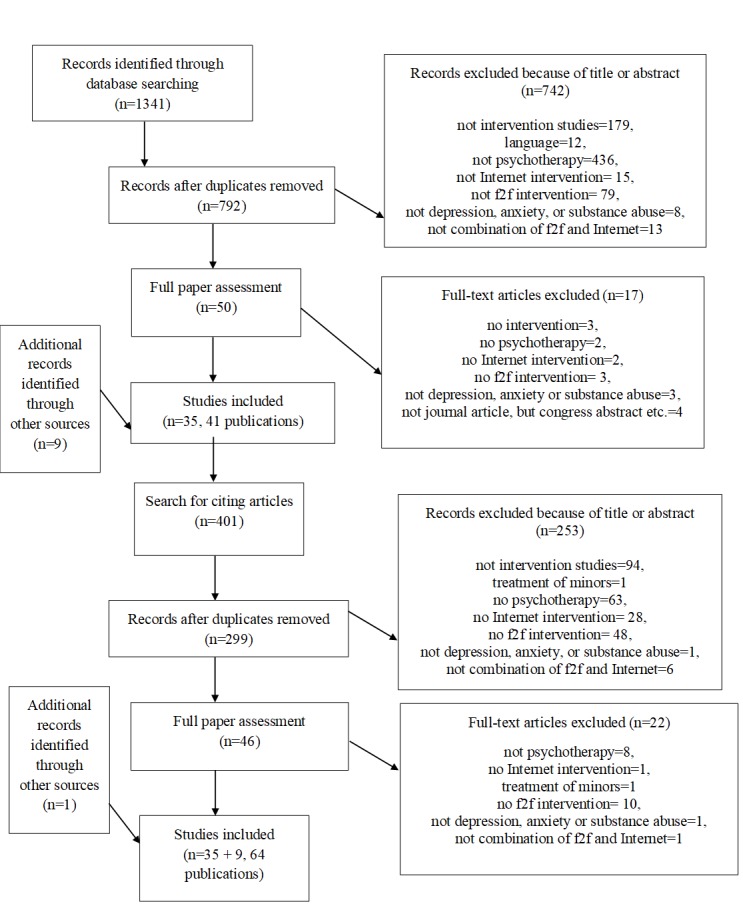
Flowchart of included and excluded studies.

See Figure 1 for a flowchart of included and excluded studies. In total, we identified 64 publications describing 44 studies. Eight of the included studies were study protocols that had not yet published results in November 2016.

Of the included studies, 27 were randomized controlled trials (RCTs), 4 were non-RCTs, 5 were pre-post studies without a control group, 4 were case studies, 3 were preliminary evaluation or acceptability studies, and 1 was a qualitative study. See Multimedia Appendix 1 for detailed study characteristics.

Of the 44 studies, 8 were rated high quality studies (18%). A control group was involved in 31 (70%, 18/44) of the studies. Twelve studies (27%, 12/44) involved a nonblended active control group with the same number of sessions as the intervention group. Only 6 studies (14%, 6/44) involved a sample of 264 or more. All relevant statistics, including effect sizes, were reported by 17 studies (39%, 17/44). See Multimedia Appendix 3 for all quality assessment scores.

Most studies were conducted or planned in the United States (n=12), followed by the Netherlands (n=9) and Germany (n=8). Six studies were conducted in Australia, four in Norway, three in the United Kingdom, and two in Sweden.

The Internet part of the studies’ blended interventions generally used Web-based programs with modules combining techniques such as cognitive, behavioral, and/or emotion-focused interventions, some of them with email support. However, there were two exceptions. One study [23] reports on using emails and Internet sites on psychoeducation and other issues to improve organization and to assist with common problems related to depression. The other study [44] reports offering weekly Internet group chats with a therapist (in open groups of 8-10 participants) to focus on problems arising in readjusting to everyday life after inpatient treatment.

### Disorders Addressed

Of the included 44 studies, 20 studies focused on treating depression only, eight focused on anxiety disorders only, and eight focused on substance abuse only. One study described the treatment of comorbid depression and substance abuse; three studies treated both depression and anxiety. The remaining four studies had a transdiagnostic concept involving depression, anxiety, and other mental disorders. See Multimedia Appendix 1 for details on disorders addressed.

### Concept of Blended Care and Stage of Treatment

Following the study selection process, we clustered the studies into the following types of blended care (Multimedia Appendix 1):

1. Integrated blended interventions with face-to-face focus: These blended interventions are based on an face-to-face intervention that is complemented or partly replaced by an Internet intervention; face-to-face and Internet-based elements are provided within the same period.
2. Integrated blended interventions with Internet focus: These blended interventions are based on Internet interventions that are partly replaced or complemented by face-to-face sessions; face-to-face and Internet-based elements are provided within the same period.
3. Sequential blended interventions with Internet, then face-to-face: These blended interventions arrange the Internet intervention part before the face-to-face treatment, such as within stepped care.
4. Sequential blended interventions with face-to-face, then Internet: These blended interventions arrange the Internet intervention part after the face-to-face treatment as in an aftercare program.

The majority of studies (n=29) used a concept of integrated blended intervention, with face-to-face and Internet-based elements being provided within the same period. Among those, 18 studies focused on the face-to-face intervention, considering the Internet intervention as a replacement of some of the face-to-face sessions or as an adjunct, whereas 11 studies focused on the Internet intervention as the basis of treatment where the face-to-face sessions served as an adjunct, for example, for increasing adherence to the Internet-based modules.

The remaining 15 studies presented sequential blended interventions, arranging the Internet intervention part before or following the face-to-face treatment. Nine of them placed the Internet intervention before the face-to-face treatment either as part of a stepped or matched care program (n=4) or for bridging waiting time for referrals on waiting lists for face-to-face psychotherapy (n=5). Six studies placed the Internet intervention after the face-to-face treatment as an aftercare concept.

### Aims

**Figure 2:**
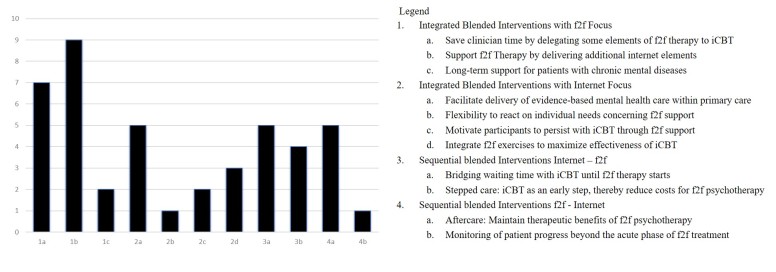
Aims of blended therapy.

The studies’ aims for choosing to use a blended intervention can be classified by the concepts that were used (see Multimedia Appendix 1 for details). As some studies mentioned various aims, we clustered the interventions by their main objective. See Figure 2 for a summary of aims of blended therapy.

#### Integrated Blended Interventions With Face-to-Face (F2F) Focus

Among the 18 integrated blended interventions with face-to-face focus, seven aimed at delegating some elements of face-to-face therapy to Internet-based cognitive behavioral therapy (iCBT) and thereby, saving clinician time and reducing overall costs [15,51,54,58,62,69,75]. Nine of them aimed at supporting face-to-face therapy by delivering additional Internet elements and thereby increasing effectiveness of face-to-face psychotherapy [23-25,37,40,55,60,61,77,78]. Two studies stated as their aim integrating Internet elements with face-to-face psychotherapy to establish a proactive and long-term approach to the management of chronic conditions, thus providing long-term support for patients with chronic or recurrent mental diseases beyond the acute phase of face-to-face treatment [59,68].

#### Integrated Blended Interventions With Internet Focus

Five of the 11 integrated blended interventions with an Internet focus aimed at improving the delivery of evidence-based treatment in primary care [31,48,50,65,72], for instance, by assisting GPs in providing evidence-based mental health care programs [65]. Three of the studies aimed at integrating face-to-face sessions to maximize the effectiveness of iCBT [19,64,70]. Other aims of these studies were to offer an Internet intervention to all participants with complementary face-to-face sessions as needed by the individual participant and as such to increase flexibility to meet the needs of different clients concerning face-to-face support [79], or, through face-to-face support, to motivate participants to persist with iCBT [45,74].

#### Sequential Blended Interventions With Internet, Then F2F

The nine sequential blended interventions that started with the Internet intervention either aimed at bridging waiting time (n=5) with iCBT until face-to-face therapy started [53,57,67,73,80] or worked with a blended stepped care concept, aiming at delivering low-threshold iCBT as an early step and thereby reducing costs for subsequent face-to-facepsychotherapy (n=4) (by reducing either the number of v sessions or the number of patients treated in the subsequent face-to-facephase) [20,26,46,47].

#### Sequential Blended Interventions With F2F, Then Internet

Five of the six sequential blended interventions that started with the face-to-face intervention were designed as aftercare programs aiming at maintaining therapeutic benefits of face-to-faceresidential or inpatient psychotherapy through subsequent iCBT [22,41,44,56,76]. Another study aimed to monitor patient progress beyond the acute phase of face-to-face treatment for a long time and to maintain the therapeutic relationship in the absence of face-to-face contacts in a remote setting [71].

### Outcome

See Multimedia Appendix 2 for details. Given the variety of different study types (eg, study protocols, case studies, and qualitative studies) and that outcome measures, study designs, and aims differ substantially, it is not currently possible to summarize the effects using meta-analytic techniques.

Of the 44 identified studies, eight were study protocols and 36 had been completed. Among the 36 completed studies, 23 involved a control group. Out of the 23 completed studies with a control group, four studies compared the blended intervention with no treatment, for instance, a waiting-list group [19,41,44,48]. Nine studies compared the blended intervention group with a nonblended face-to-face intervention with an equal amount or more sessions [24,32,50,53-55,57,62,75], six studies compared the blended intervention with treatment-as-usual without controlling the number of sessions [20,26,32,37,40], and two studies compared the blended intervention with a nonblended Internet intervention [64,65]. The remaining two studies involved both Internet-based and face-to-face nonblended control groups [55,70].

Among the eight study protocols, two studies compared the blended intervention with TAU without controlling for the number of sessions [46,51,59], whereas four studies compared the blended intervention-group with a nonblended face-to-face intervention with an equal number or more sessions [15,53,60,69]. One study compared the blended intervention (in that case, aftercare after inpatient treatment) with a placebo (inpatient treatment plus access after discharge to an Internet platform with information on depression [76]), and one study compared it with no treatment [67].

**Figure 3:**
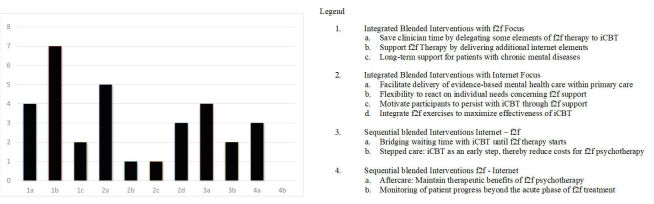
Aims of blended therapy of studies with published outcome.

Although cost-effectiveness or cost-savings was in some way considered by almost all 44 studies, only three of the completed studies elaborated on it and evaluated potential cost-effectiveness or cost-savings [33,52,81]. However, three additional study protocols claimed to be planning to evaluate cost-effectiveness [15,46,51,69]. See Figure 3 for a summary of aims of blended therapy in completed studies.

#### Integrated Blended Interventions With F2F Focus

Of the six studies that were delegating some elements of face-to-face therapy to iCBT, three studies were able to show that, by doing that, 50% to 86 % of clinician time could be saved without reducing the therapeutic outcome of depression and anxiety treatment [54,62,75]. The other three were study protocols [15,51,69].

Six of the nine studies aiming at supporting face-to-face therapy by delivering Internet elements were able to show that adding Internet elements can lead to lower dropout rates and/or greater abstinence rates of patients with substance abuse compared with stand-alone face-to-face interventions [24,40,55,77]. One study had a pre-post design [61] and showed that its blended concept led to a reduction in symptomatology that was maintained for 12 months. One was a case study where a patient diagnosed with depression as well as her therapist were able to make creative use of Web-based resources for purposes such as psychoeducation or job search [23]. One was a study protocol [60].

One study aiming at establishing a proactive and long-term approach to the management of chronic mental diseases beyond the acute phase of face-to-face treatment [68] showed large and significant pre-post effect sizes. One study with a similar aim [59] showed that an Internet-delivered adjunct to TAU may reduce the number of unwell weeks in patients with recurrent depression compared with TAU and thus, reduce the lifelong burden of depression.

#### Integrated Blended Interventions With Internet Focus

Regarding the aim of integrating face-to-face sessions into Internet interventions to maximize effectiveness of iCBT, Sethi et al [70] found the blended intervention to be superior in reducing symptoms of depression, anxiety, and/or automatic negative thoughts in comparison with three control groups: nonblended Internet intervention, nonblended face-to-faceintervention, and no intervention. One study treating depression [64] did not find a difference between the blended and the nonblended intervention, whereas a third study did not have a nonblended control group but found the blended intervention to be superior compared with a waiting-list control group [19].

Using blended interventions to improve the delivery of evidence-based treatment in primary care has been successful in acceptability and in reducing symptomatology [31,48,50,65,72]. However, two controlled studies [50,65] could not show a superiority of the blended intervention over an active nonblended control group.

In a study aiming at increasing the flexibility of an Internet intervention by offering complementary face-to-face sessions as needed by the individual participant [79], the average number of face-to-face sessions participants needed was equal to or less than 3.7, with the program still substantially and significantly reducing depression symptomatology. This was a pilot study without a control group, yet the authors cite a study that treated participants with similar pre- and post-Beck depression inventory scores as their study [82]. The other study’s participants needed an average of 11.6 face-to-face sessions for a similar symptom reduction, and the authors therefore claim that their treatment needs about eight fewer individual sessions per client.

A study aiming at motivating participants to persist with iCBT through face-to-face support [74] used a qualitative design. It found that for persistence in a blended treatment, acknowledgment may be related to flexibility and feedback from a qualified therapist in the face-to-face consultations beneath personal resources such as a sense of belonging toward partners, family, and friends. A case study with a similar aim [45] showed good adherence and symptom remission in a woman suffering from antepartum depression, who was thus able to avoid antidepressant use during pregnancy.

#### Sequential Blended Interventions With Internet, Then F2F

Of the five studies aiming at bridging waiting time with iCBT until face-to-face therapy starts, two studies [53,57] showed significant between-group effect sizes between the iCBT waiting-list group and the control waiting-list group. One study failed to show a superiority of the iCBT waiting-list group compared with a control condition with participants who remained on the wait-list with a self-help booklet [83]. One of the studies showed in a pre-post design that the Internet intervention could substantially and significantly reduce symptomatology [73]. The remaining study with this aim [67] is a study protocol.

Two of the completed studies working with a blended stepped care concept did not find a significant superiority of the blended intervention compared with face-to-face [47] or TAU [20]. Haug et al [47] even found a signiﬁcantly better outcome of the control group (nonblended f2f) in one of the outcome measures. Neither Haug et al nor Braamse et al [20] reported results on cost-saving or cost-effectiveness; however, one study protocol with a stepped care concept mentioned planning to evaluate cost-effectiveness [46].

#### Sequential Blended Interventions With F2F, Then Internet

Two of the five studies with Internet intervention designed as maintenance treatment found a substantially and significantly lower relapse rate compared with access to TAU groups [41,44]. One further study was a case study reporting how iCBT aftercare substantially reduced depression, leading to remission after discharge from inpatient treatment [22]. One study with an empirical correlational design and without a control group showed a significant relationship between the number of iCBT modules accessed and substance abuse reduction in the year following inpatient treatment when controlling for motivation, self-efficacy, and pretreatment substance abuse [56]. One study was a study protocol [76].

One case study aiming at long-term monitoring of patient progress beyond the acute phase of face-to-face treatment, as well as maintaining the therapeutic relationship in the absence of face-to-face contacts in a remote setting [71], reported continuing therapeutic contacts and a reduction from very severe depression symptoms to remission within 46 weeks.

## Discussion

### Principal Findings

This study has shown that, in the past few years, a growing number of blended interventions that combine Internet and face-to-face interventions have been developed for common mental health problems. The interventions we found have different concepts and various aims. First results are encouraging and suggest that, compared with stand-alone face-to-face interventions, blended therapy may save clinician time without reducing therapy outcome, can lead to lower dropout rates and/or greater abstinence rates of patients with substance abuse, may help maintain effects of inpatient therapy, and may even increase the effects of psychotherapy, although results are mixed and more research is clearly needed.

Compared with the field of both Internet-based stand-alone treatment and face-to-face interventions, the field of blended interventions is, however, under development and still small. Most aims of the interventions stated in the studies have not been evaluated rigorously. For instance, only 19 out of 36 completed studies were RCTs, and only eight of them ensured comparability by involving a nonblended intervention control group with the same number of sessions [27,50,57,62,70,75,78,83]. Only eight studies were considered high quality studies, and only six studies involved a control group big enough to detect effect sizes of *d*=0.35 or less. Also, the cost-effectiveness of blended treatments compared with face-to-face psychotherapy has only been evaluated in three out of the 36 completed studies. However, this issue is focused upon in current large research projects [46,84].

Several questions remain. For instance, not much is known about the optimal ratio of Internet and face-to-facesessions that would allow costs to be minimized while maintaining or increasing effectiveness. Only one study in our review is moving toward answering this question: In the study by Jacmon et al [79], clients received face-to-face sessions as wished as an adjunct to iCBT. Also, clients were actively invited by therapists to undertake face-to-face contacts if a depression measure indicated that depression continued at clinical levels. Jacmon et al reported that this intervention produced effects at least as large as a completely face-to-face treatment, with an average of about eight fewer individual sessions per client in comparison with a similar nonblended face-to-face treatment in another study. However, the findings of this pilot study cannot be generalized because of its small sample size and lack of a control group. Another open question is which elements of face-to-face therapy can most suitably be delegated to the Internet. Possibly, elements that do not need an intensive dialogue between client and therapist, such as psychoeducation, may most easily be delegated to the Internet [16]. Also, it remains unclear who would benefit most from which relationship between face-to-face and Internet modules. For instance, patients who have difficulty in expressing all of their thoughts and feelings in writing (eg, because of lack of introspection or because they are not conscious of their own role in maintaining their disorder) might need more elements of face-to-face intervention psychotherapy. This might also be the case for patients with severe, chronic, or personality disorders. A helpful tool to set up a personalized blended treatment taking the patients’ characteristics into account might be the recently developed “Fit for Blended Care” instrument [17].

Moreover, although a number of studies stated that increasing the effectiveness of face-to-face psychotherapy is an aim of the blending of treatments with Internet options, evidence for this hypothesis is limited to four studies with an integrated concept [24,25,32,70]. Such an assumption is, however, supported by a recent meta-analysis by Lindhiem and colleagues [85]. On the basis of 10 RCTs, they found that, in comparison with strictly on-site interventions, psychological interventions were considerably more effective for a range of conditions when behavior changes between face-to-face sessions had been supported by a mobile component, such as short message service (SMS; *d*=0.27). There is also some evidence that telephone-based interventions may help maintain advances achieved during outpatient treatment or increase effectiveness of face-to-face interventions [86,87]. Nevertheless, more research is clearly needed to conclude whether the effectiveness of psychotherapy can potentially be increased by using blended treatments. Besides the identified advantages of blended interventions, for example, saving clinician time, improving success rates in the reduction of symptomatology, and helping to prevent relapse after face-to-face therapy, these interventions might possibly have limitations compared with face-to-face interventions. For instance, in the study by Marks et al [62], dropout was substantially higher in the mainly Internet-based group compared with the face-to-face control group (43% if mainly computer-guided and 24% if entirely clinician-guided). On the other hand, other studies have reported similar [50,75] or even lower [24] dropout rates of a blended compared with a nonblended intervention. Yet, the questions of blended interventions’ negative effects and potential losses compared with nonblended interventions remain to be answered, just as much as the one about potential negative effects of Internet-based treatments [12].

An interesting question we encountered is what blended treatments exactly entail. The identified studies in our review that explicitly use the term “blended” are the more recently published ones and describe integrated blended treatments, that is, combined treatments that provide face-to-face and Internet-based elements within the same period. Although there is currently no clear definition, this description might be an implicit understanding of blended interventions that is more limiting than our definition. We consider that blended interventions should also include the combination of the Internet-based interventions arranged before or after the face-to-face treatments following clear rules and procedures, such as within stepped care or aftercare that directly follows the acute phase treatment. In light of the numerous studies describing sequential blended interventions, we decided to use this wider definition.

### Limitations

This study has some limitations. As within every systematic review, the risk of selection bias when including relevant studies needs to be considered. However, our use of independent ratings by two of the authors worked against this bias. In addition, publication bias needs to be considered. We did not contact authors for additional data or additional studies, which would have automatically limited the number of studies that could be included. Also, we only reviewed bibliographies from included studies, so we possibly missed studies that were cited in papers that we reviewed but did not include. Furthermore, the types of studies we included were heterogeneous (for instance, we included study protocols). It is possible that more narrow inclusion criteria (eg, randomized controlled studies with nonblended active control groups only) would have produced more information about the effectiveness of blended interventions. However, the number of such studies in the field is yet very small, and future studies are needed to explore whether blended treatments can, for example, be superior compared with nonblended treatments with regard to effect sizes or lower costs.

For further research, it would be of interest to explore effectiveness and cost-effectiveness of blended concepts, especially concerning the optimal balance of face-to-face and Internet interventions. Information on this aspect would help determine which therapy with which theoretical foundation (such as cognitive behavioral therapy or psychoanalysis) is feasible for blended interventions, as well as where the optimal balance of therapy modules lies for individual patients in light of the type and severity of disorder, state of motivation, ability of introspection, and other variables such as age, gender, and computer skills.

### Conclusions

To conclude, we have found that several studies have shown that blended interventions are feasible and effective compared with no treatment controls. There are many different kinds of blended concepts that, in every phase of treatment, may offer added value concerning either effectiveness or cost-effectiveness. However, to evaluate the actual benefit of blended concepts for mental health care, more RCTs on effectiveness and cost-effectiveness compared with traditional nonblended psychotherapy are required. Thus, more research is needed, especially concerning disorders for which blended interventions are particularly effective, the amount of face-to-face contact that is needed, and the parts of therapy that can be delegated to the Internet.

## Appendix

Multimedia Appendix 1 Previous psychological research combining Internet and face-to-face psychotherapy.

Multimedia Appendix 2 Outcome of previous psychological research combining Internet and face-to-face (f2f) psychotherapy.

Multimedia Appendix 3 Study quality assessment of blended treatments.

## Abbreviations

f2f: face-to-face
RCTs: randomized controlled trials
