# Supplementary material for: Blending Face-to-Face and Internet-Based Interventions for the Treatment of Mental Disorders in Adults: Systematic Review
Source: J Med Internet Res. 2017 Sep 15;19(9):e306. doi: 10.2196/jmir.6588 (PMC5622288; doi:10.2196/jmir.6588)
Supplement: Multimedia Appendix 1 [file jmir_v19i9e306_app1.pdf]

Table 2. Previous psychological research combining Internet and face-to-face (f2f) psychotherapy.

Study (year), country	Condition	Design	Study conditions	N	Classification of blended concept	Aim of blended therapy
Anderson et al (2016), Sweden [43]	Anxiety	2-arm randomized controlled trial (RCT)	Intervention group (IG): 9 weeks Internet-based cognitive behavioral therapy (iCBT) with email support + 2 face-to-face (f2f) 3-hour group exposure sessions<br><br>Control group (CG): waiting list	IG: N=32<br><br>CG: N=32	Integrated blended intervention with Internet focus	2d
Braamse et al (2010, 2016), Netherlands [54,74]	Transdiagnostic	2-arm RCT	IG: 30 weeks stepped care:<br>1. watchful waiting,<br>2. iCBT self-help<br>3. f2f psychotherapy<br><br>CG: 30 weeks treatment-as-usual (TAU)	IG: N=50<br><br>CG: N=49	Sequential blended intervention Internet—f2f	3b
Brakemeier et al (2013), Germany [58]	Depression	Case study	3-month step-down Internet intervention (CBASP@home); 9 times Internet-based situational analysis after inpatient treatment with feedback from inpatient therapist	N=2	Sequential blended intervention f2f—Internet	4a
Callan et al (2009), United States	Depression	Case study	Use of Internet sites and emails to improve medication and therapy homework adherence,	N=1	Integrated blended intervention	1b

[19]			time management, journaling, and nutrition monitoring		with f2f focus	
Campbell et al (2012, 2014, 2015), Cochran et al (2015), Cunningham et al (2015), Murphy et al (2016), Tofighi et al (2016), United States [29,31,56,69, 75-77]	Substance abuse	2-arm RCT	IG: 12 weeks outpatient TAU + iCBT (Therapeutic education system [TES], 62 interactive multimedia modules), iCBT substituting for about 2 hours of standard care per week<br><br>CG: 12 weeks TAU: at least two f2f therapeutic group or individual sessions per week, lasting at least 2 hours	IG: N=255<br><br>CG: N=252	Integrated blended intervention with f2f focus	1b
Campbell et al (2015), United States [41]	Substance abuse	Mixed-method acceptability study	8 weeks outpatient TAU + iCBT using on-site computers (TES, 32 interactive multimedia modules), four modules per week at two different visits during the week, visits usually linked to patients' attendance at TAU	N=40	Integrated blended intervention with Internet focus	2a
Carroll et al (2008, 2009, 2011), Sugarman et al (2010), Olmstead et al (2010), United States [63,65,78-80]	Substance abuse	2-arm RCT	IG: 8 weeks TAU + biweekly iCBT (computer-based training for cognitive behavioral therapy, CBT4CBT, 6 modules)<br><br>CG: 8 weeks TAU (weekly individual and group sessions of general drug	IG: N=39<br><br>CG: N=38	Integrated blended intervention with f2f focus	1b

			counseling)			
Carroll et all (2014, 2015), Morie et al (2015), United States [34,81,82]	Substance abuse	2-arm RCT	IG: 8 weeks TAU + biweekly iCBT (CBT4CBT, 7 modules)<br><br>CG: 8 weeks TAU (weekly individual and group sessions of general drug counseling)	IG: N=47<br><br>CG: N=54	Integrated blended intervention with f2f focus	1b
Christensen et al (2014), United States [32]	Substance abuse	2-arm RCT	IG: 12 weeks TAU + iCBT, 3 times per week, approximately 30 min per visit, 69 modules presented on clinic computers<br><br>CG: 12 weeks TAU (contingency management + biweekly 30 min f2f + buprenorphine dosing)	IG: N=92<br><br>CG: N=78	Integrated blended intervention with f2f focus	1b
Ebert et al (2013), Germany [59,83,84]	Transdiagnostic	2-arm RCT	IG: 12 weeks access to TAU + iCBT (Transdiagnostic Internet-based maintenance treatment, with self-management module, asynchronous patient-coach communication, Internet patient support group) after inpatient treatment<br><br>CG: Access to TAU	IG: N=200<br><br>CG: N=200	Sequential blended interventions f2f—Internet	4a
Golkaramnay et al (2007), Germany	Transdiagnostic	Pre-post + control	IG: 12-15 weeks Internet group chat with a therapist for 90 min in open groups of 8-10	IG: N=114	Sequential blended interventions f2f—Internet	4a

[20]			participants after discharge of inpatient treatment + access to TAU<br><br>CG: Access to TAU	CG:<br>N=114		
Hantsoo et al (2014),<br>United States<br>[48]	Depression	Case study	Eight therapy sessions over 6 weeks with a clinical psychologist (3.75 hours f2f), between-session Internet-based modules (“Good Days Ahead”)	N=1	Integrated blended intervention with Internet focus	2c
Härter et al (2015), Germany<br>[57]	Depression	2-arm RCT<br>(study protocol)	IG: Stepped care:<br>1. watchful waiting<br>2. bibliotherapy or iCBT self-help or telephone-based psychotherapy<br>3. psychotherapy or pharmacotherapy<br>4. psycho- and<br>pharmacotherapy combined (inpatient or outpatient setting)<br><br>CG: TAU	IG:<br>N=660<br><br>CG:<br>N=200	Sequential blended interventions Internet—f2f	3b
Haug et al (2015), Norway<br>[55]	Anxiety	2-arm RCT	IG: Stepped care:<br>1. psychoeducation<br>2. iCBT<br>3. f2f-CBT<br><br>CG: Immediate f2f-CBT	IG:<br>N=85<br><br>CG:<br>N=88	Sequential blended interventions Internet—f2f	3b
Høifødt et al (2013, 2015), Norway	Depression	2-arm RCT	IG: 7 weeks iCBT with 15-30 min f2f support after each module	IG:<br>N=52	Integrated blended intervention	2a

[38,85]			CG: Access to TAU	CG: N=54	with Internet focus	
Jacmon et al (2019) Australia [4]	Depression	Pre-post	4-6 weeks Internet course on CBT skills with weekly email support, arrangement of individual f2f sessions as needed; average 3.7 f2f sessions	N=9	Integrated blended intervention with Internet focus	2b
Kay-Lambkin et al (2011), Australia [39]	Comorbid depression + substance abuse	3-arm RCT	IG: Blended: 1 initial f2f session + 9 sessions iCBT with therapist support<br>CG1: f2f: 10 sessions f2f CBT<br>CG 2: Brief intervention (BI), one individual f2f session	IG: N=32<br>CG1: N=35<br>CG 2: N=30	Integrated blended intervention with Internet focus	2a
Kemmeren et al (2016), Netherlands [25,64]	Depression	2-arm RCT (study protocol)	IG: 19-20 weeks weekly alternating 45 min f2f sessions and iCBT sessions<br>CG: TAU	IG: N=78<br>CG: N=78<br>-	Integrated blended intervention with f2f focus	1a
Kenter, Warmerdam et al (2013), Netherlands [50]	Transdiagnostic	Pre-post + control	IG: 5 weeks Internet problem-solving treatment with email feedback; subsequently f2f psychotherapy<br>CG: 5 weeks waiting list, then f2f psychotherapy	IG: N=55<br>CG: N=49	Sequential blended interventions Internet—f2f	3a

Kenter et al (2013, 2016), Kolovos et al (2016) Netherlands<br><br>[49,64,68)	Depression	2-arm RCT	IG: 5 weeks Internet problem-solving treatment with email feedback; subsequently f2f psychotherapy<br><br>CG: 5 weeks waiting list + self-help problem-solving booklet, then subsequently f2f psychotherapy	IG:<br>N=136<br><br>CG:<br>N=133	Sequential blended interventions Internet—f2f	3a
Kenwright et al (2001), United Kingdom<br><br>[21]	Anxiety	Pre-post + control	IG: 4 sessions of 40 min iCBT (FearFighter) with 20 min f2f support<br><br>CG: 8 sessions f2f CBT	IG:<br>N=54<br><br>CG<br>N=31	Integrated blended intervention with f2f focus	1a
Kiluk et al (2016), United States<br><br>[35]	Substance abuse	3-arm RCT	IG: 8 weeks TAU + biweekly iCBT (computer-based training for cognitive behavioral therapy, CBT4CBT, 7 modules of 45 min)<br><br>CG1: 8 weeks TAU (weekly individual or group psychotherapy sessions)<br><br>CG2: 8 weeks on-site iCBT with brief weekly f2f monitoring	IG:<br>N=22<br><br>CG1:<br>N=22<br><br>CG2:<br>N=24	Integrated blended intervention with f2f focus	1b

Klein et al (2012), United States [60]	Substance abuse	Empirical correlational study	Internet-based disease management program 18 months following discharge from treatment, including 7 modules iCBT, opportunities for fellowship with other recovering individuals, therapist support over email and telephone	N=1124	Sequential blended interventions f2f—Internet	4a
Kok et al (2014), Netherlands [53]	Anxiety	2-arm RCT	IG: 5 weeks iCBT exposure therapy with weekly student support; subsequently f2f psychotherapy<br><br>CG: 5 weeks waiting list + self-help exposure book, subsequently f2f psychotherapy	IG: N=105<br><br>CG: N=107	Sequential blended interventions Internet—f2f	3a
Kooistra et al (2014), Netherlands [15]	Depression	2-arm RCT	IG: blended CBT (10 weeks), ten 45-min f2f sessions and nine Internet sessions<br><br>CG: CBT f2f (20 weeks), twenty 45-min sessions of f2f CBT	IG: N=75<br><br>CG: N=75	Integrated blended intervention with f2f focus	1a
Kooistra et al (2016), Netherlands [26]	Depression	Preliminary evaluation study	10 weeks 10 sessions f2f (45 min) + 10 sessions iCBT	N=9	Integrated blended intervention with f2f focus	1a
Kordy et al (2016), Germany [37]	Depression	3-arm RCT	IG1: 12-month TAU + iCBT (SUMMIT, supportive monitoring and depression management over the Internet)	IG1: N=77<br><br>IG2: N=79	Integrated blended intervention with f2f focus	1c

			IG2: 12-month TAU + iCBT (SUMMIT-PERSON: SUMMIT + clinician-guided individual + group chats)<br><br>CG: TAU including maintenance antidepressant medication and clinical management + individual crisis management plan (CMP) + project website_	CG:<br>N=80		
Krieger et al (2014), Germany<br>[28]	Depression	2-arm RCT	IG: 12 weeks regular psychotherapy (weekly hourly sessions) + iCBT<br><br>CG: 12 weeks regular psychotherapy (weekly hourly sessions)	IG:<br>N= 400<br><br>CG:<br>N=400	Integrated blended intervention with f2f focus	1b
Månsson et al (2013), Sweden<br>[27]	Depression and anxiety	Pre-post	Blended f2f and iCBT, 8-9 weeks	N=15	Integrated blended intervention with f2f focus	1b
Marks et al (2014), United Kingdom<br>[22]	Anxiety	3-arm RCT	IG: Blended: 6-hour-long iCBT sessions over 10 weeks, f2f support: up to 5 min at beginning, up to 15 min at the end<br><br>CG1: f2f: 6-hour-long individual treatment sessions over 10 weeks f2f CBT	IG:<br>N=37<br><br>CG1:<br>N=39<br><br>CG2:<br>N=17	Integrated blended intervention with f2f focus	1a

			CG 2: blended attention control: 6-hour-long sessions over 10 weeks guidance in self-relaxation techniques by a computer, f2f support: up to 5 min at beginning, up to 15 min at the end			
Marsch et al (2016), Kim et al (2016), United States [86]	Substance abuse	2-arm RCT	IG: 52 weeks outpatient TAU + iCBT (TES, 62 interactive multimedia modules), iCBT substituting for about 30 min of standard care per week, 2 modules of about 15 min per session<br><br>CG: 52 weeks TAU: daily methadone maintenance doses, weekly or biweekly individual counseling sessions of up to 60 min	IG: N=80<br><br>CG: N=80	Integrated blended intervention with f2f focus	1b
Nordmo et al (2015), Norway [44]	Anxiety	2-arm RCT	IG: 90 min session f2f + nine iCBT modules, weekly 10-min therapist support by phone<br><br>CG: nine iCBT modules, weekly 10-min therapist support by phone, no f2f	IG: N=17<br><br>CG: N=20	Integrated blended intervention with Internet focus	2d
Pier et al (2008), Shandley et al (2008), Australia	Anxiety	Pre-post + control	IG:12 weeks iCBT (Panic online, PO) with f2f support by a general practitioner (GP) every 1-2 weeks	IG: N=53	Integrated blended intervention with Internet focus	2a

[40,87]			CG: 12 weeks iCBT only (email support every 1-2 weeks)	CG:<br>N=43		
Reins et al (2013), Germany<br>[51]	Depression	2-arm RCT<br>(study protocol)	IG: About 3 weeks access to TAU + six iCBT modules, then inpatient treatment<br>CG: Access to TAU, then inpatient treatment	N=200	Sequential blended interventions Internet—f2f	3a
Robertson et al (2006), Australia<br>[36]	Depression	Pre-post	12 iCBT sessions (RecoveryRoad) with Internet progress monitoring over approximately 12 months as adjunct to usual treatment, clinicians had Internet access to patients' progress monitoring outcomes	N=144	Integrated blended intervention with f2f focus	1c
Romijn et al (2015), Netherlands<br>[23]	Anxiety	2-arm RCT	IG: 15 CBT sessions, with weekly alternating 45-min f2f sessions and Internet sessions<br>CG: 15 weekly 45-min f2f CBT sessions	IG:<br>N=78<br>CG:<br>N=78	Integrated blended intervention with f2f focus	1a
Sethi et al (2010), Australia<br>[45]	Depression, anxiety	4-arm RCT	IG: Blended: five CBT sessions over 3 weeks, first half f2f CBT, second half iCBT (MoodGYM)<br>CG1: f2f: five CBT sessions over 3 weeks	IG:<br>N=9<br>CG1:<br>N=10<br>CG2:	Integrated blended intervention with internet focus	2d

			CG2: iCBT: five MoodGYM sessions over 3 weeks<br><br>CG3: No intervention	N=10<br><br>CG3:<br>N=9		
Tannenbaum and Spiranovic (2010), Australia<br><br>[62]	Depression	Case study	Continuous care provided over more than 5 years by Dr Tannenbusch: first f2f CBT + medication, partly inpatient, later remote treatment: medication prescribed by GP, monitored by Dr Tannenbusch + iCBT (health steps program)	N=1	Sequential blended interventions f2f—Internet	4b
Van Straren et al (2010), Netherlands<br><br>[56]	Depression and anxiety	2-arm RCT<br><br>(study protocol)	IG: Stepped care:<br>1. watchful waiting<br>2. iCBT or CBT book self-help<br>3. brief f2f (5-7 sessions of 30-45 min each)<br>4. longer f2f and/or medication<br><br>CG: TAU	IG: N=100<br><br>CG: N=100	Sequential blended interventions Internet—f2f	3b
Van Voorhees et al (2007), United States<br><br>[42]	Depression	Preliminary evaluation study	iCBT (11 modules) + 2 f2f sessions in primary care (one initial primary care motivational interview, one follow-up motivational interview)	N=14	Integrated blended intervention with Internet focus	2a
Whitfield et al (2006),	Depression	Pre-post	Six hourly sessions iCBT, some f2f support	N=78	Sequential blended	3a

United Kingdom<br><br>[52]			from a self-help support nurse, one session per week		interventions Internet—f2f	
Wilhelmsen et al (2013), Norway<br><br>[47]	Depression	Qualitative study	Five iCBT modules, f2f support sessions of 20-30 min between modules	N=15	Integrated blended intervention with Internet focus	2c
Wright et al (2005), United States<br><br>[24]	Depression	3-arm RCT	IG: 8 weeks of iCBT with f2f support; 9 sessions with 25-min f2f<br><br>CG1: 8 weeks f2f CBT, 9 sessions of 50 min<br><br>CG2: waiting list	IG: N=15<br><br>CG1: N=15<br><br>CG2: N=15	Integrated blended intervention with f2f focus	1a
Zwerenz et al (2015), Germany<br><br>[61]	Depression	2-arm RCT<br><br>(study protocol)	IG: Inpatient psychotherapy + 12-week Internet-based self-help treatment (deprexis), 12 modules, beginning during inpatient treatment<br><br>CG: Inpatient psychotherapy + 12 weeks access to an Internet platform with weekly updated information on depression	IG: N=120<br><br>CG: N=120	Sequential blended interventions f2f—Internet	4a

Code Scheme

1. Integrated blended interventions with f2f focus
   a. Delegating of some elements of f2f therapy to Internet-based cognitive behavioral therapy (iCBT) and thereby save clinician time and reduce overall cost

   b. Supporting f2f therapy by delivering additional Internet elements and thereby increase effectiveness of f2f psychotherapy

   c. Providing long-term support for patients with chronic mental diseases

2. Integrated blended interventions with Internet focus
   a. Facilitate delivery of evidence-based mental health treatments within primary care (eg, assist general practitioners (GPs) to provide evidence-based mental health care)
   b. Flexibility to react on individual needs concerning f2f support
   c. Motivate participants to persist with iCBT through f2f support
   d. Integrate f2f exercises to maximize effectiveness of iCBT
3. Sequential blended interventions Internet—f2f
   a. Bridging waiting time with iCBT until f2f therapy starts
   b. Stepped care approach: deliver low-threshold iCBT as an early step, thereby reducing costs for subsequent f2f psychotherapy
4. Sequential blended interventions f2f—Internet
   a. Aftercare: maintain therapeutic benefits of f2f psychotherapy through subsequent iCBT
   b. Monitoring of patient progress beyond the acute phase of f2f treatment
