# Supplementary material for: Blending Face-to-Face and Internet-Based Interventions for the Treatment of Mental Disorders in Adults: Systematic Review
Source: J Med Internet Res. 2017 Sep 15;19(9):e306. doi: 10.2196/jmir.6588 (PMC5622288; doi:10.2196/jmir.6588)
Supplement: Multimedia Appendix 2 [file jmir_v19i9e306_app2.pdf]

Table 4. Outcome of previous psychological research combining Internet and face-to-face (f2f) psychotherapy.

Study (year), country	Inclusion criteria	Design	Study conditions	N	(Primary) outcome measures	Results
Andersson et al (2006), Sweden<br><br>[43]	Diagnostic and Statistical Manual of Mental Disorders, 4th Edition (DSM-IV) diagnosis of social phobia according to the Social Phobia Screening Questionnaire (SPSQ)	2-arm RCT	Intervention group (IG): 9 weeks Internet-based cognitive behavioral therapy (iCBT) with email support + 2 face-to-face (f2f) 3-hour group exposure sessions<br><br>Control group (CG): waiting list	IG:<br>N=32<br><br>CG:<br>N=32	Self-report version of the Liebowitz Social Anxiety Scale (LSAS-SR); Social Phobia Scale (SPS), Social Interaction Anxiety Scale (SIAS), SPSQ, and Personal Report on Confidence as a Speaker (PRCS)	mean d_{within}= 0.87<br><br>mean $d_{between}$= 0.70
Braamse et al (2016), Netherlands<br><br>[54]	Diagnosis of hematological malignancy	2-arm RCT	IG: 30 weeks stepped care:<br>1. watchful waiting,<br>2. iCBT self-help<br>3. f2f psychotherapy<br><br>CG: 30 weeks treatment-as-usual (TAU)	IG:<br>N=50<br><br>CG:<br>N=49	Hospital Anxiety and Depression Scale (HADS), European Organization for Research and Treatment of Cancer Quality of Life Questionnaire-C30 (EORTC QLQ-C30)	Psychological distress (HADS total score)<br>$d_{between}$=0.11 not significant (ns)<br>Depression (HADS subscale)<br>$d_{between}$=0.01 ns<br>Anxiety (HADS subscale)<br>$d_{between}$=0.19 ns<br>Physical functioning (EORTC QLQ-C30)<br>$d_{between}$=−0.02 ns
Campbell et al (2015), United States<br><br>[41]	Self-identification as American Indians or Alaska natives within the first 30 days	Mixed-method acceptability study	8 weeks outpatient TAU + iCBT using on-site computers (Therapeutic Education System [TES], 32 interactive multimedia modules),	N=40	Proportion of participants agreeing to participate, number of modules	59 % (40/68) of approached clients enrolled, all but 3 participants completed at least one module, among those

	of treatment episode in one of two urban outpatient substance abuse treatment programs		four modules per week at two different visits during the week, visits usually linked to patients' attendance at TAU		completed, participant feedback survey, semistructured interview 1 week after treatment	that completed modules, the mean number was 18.6 (standard deviation [SD]=9.2) TES acceptable across seven indices (range 7.8-9.4). Qualitative interviews: adaptation specific to American Indians or Alaska natives culture could improve adoption
Campbell et al (2012, 2014, 2015), Cochran et al (2015), Cunningham et al (2015), Murphy et al (2016), Tofighi et al (2016), United States [29,31,66,69, 75-77]	Self-report of illicit substances use in the 30 days before study entry, or 60 days for those exiting a controlled environment	2-arm RCT	IG: 12 weeks outpatient TAU + iCBT (TES, 62 interactive multimedia modules), iCBT substituting for about 2 hours of standard care per week CG: 12 weeks TAU: at least two f2f therapeutic group or individual sessions per week, lasting at least 2 hours	IG: N=255 CG: N=252	Abstinence from drugs and heavy drinking (twice-weekly urine drug screens + self-report), time to drop out from treatment	IG: Lower dropout rate + greater abstinence rate compared with CG; effect more pronounced among patients with positive urine drug or breath alcohol screen at study entry (N=228), Acceptability positively associated with abstinence but only among women (*P*=.01), IG stimulant users significantly more likely to be abstinent in the final 4 weeks of treatment compared with CG, adjusted odds ratios for alcohol and cannabis) of similar magnitude but ns, abstinence among primary opioid users not improved by TES.

						Treatment condition not associated with retention<br><br>TES + TAU has at least a 95% chance of being considered cost-effective for providers and payers with willingness-to-pay thresholds as low as US $20,000 per abstinent year
Carroll et al (2008, 2009, 2011), Sugarman et al (2010), Olmstead et al (2010), United States<br><br>[63,65,78-80]	DSM-IV criteria for any current substance dependence disorder	2-arm RCT	IG: 8 weeks TAU + biweekly iCBT (computer-based training for cognitive behavioral therapy, CBT4CBT, 6 modules)<br><br>CG: 8 weeks TAU (weekly individual and group sessions of general drug counseling)	IG: N=39<br><br>CG: N=38	Urine toxicology screens, frequency<br><br>of substance use (self-report);<br><br>Balloon Analogue Risk Task (BART);<br><br>Incremental cost-effectiveness ratios (ICERs) and cost-effectiveness acceptability curves (CEACs)	Positive urine specimens submitted:<br><br>$d_{between}$=0.59[a]<br><br>Self-report: longest continuous abstinence from all drugs or alcohol<br><br>$d_{between}$=0.45 ns<br><br>Self-report: percent days abstinent from all drugs or alcohol<br><br>$d_{between}$=0.28 ns,<br><br>6-month follow-up: CG increased drug use, IG tended to improve slightly;<br><br>BART: higher levels of risk taking associated with fewer completed sessions and homework assignments and poorer substance use outcomes;<br><br>coping strategy use predictive of decrease

						in drug use, especially for participants who received CBT4CBT;<br><br>TAU + CBT4CBT likely to be cost-effective when the threshold value to decision makers of an additional drug-free specimen is greater than approximately US $21 (US $15) from the clinic (patient) perspective
Carroll et al (2014, 2015), Morie et al (2015) United States<br><br>[34,81,82]	DSM-IV criteria for current cocaine dependence disorder, stabilized on methadone (same dose for more than 2 months)	2-arm RCT	IG: 8 weeks TAU + biweekly iCBT (computer-based training for cognitive behavioral therapy, CBT4CBT, 6 modules)<br><br>CG: 8 weeks TAU (weekly individual and group sessions of general drug counseling)	IG:<br>N=47<br><br>CG:<br>N=54	Urine toxicology screens, frequency<br><br>of substance use (self-report);<br><br>saliva samples from which deoxyribonucleic acid (DNA) was extracted	Percent cocaine-free urine samples<br><br>$d_{between}$=0.59[a]<br><br>Percent drug-free urine samples<br><br>$d_{between}$=0.65[a]<br><br>Percent days of abstinence, self-report<br><br>$d_{between}$=0.30 ns<br><br>(Individuals completing treatment);<br><br>Val carriers and patients with higher baseline scores on the Toronto Alexithymia Scale (TAS) responded particularly well to CBT4CBT
Christensen et al (2014), United States<br><br>[32]	DSM-IV criteria for opioid dependence, Food and Drug Administration (FDA) qualification	2-arm RCT	IG: 12 weeks TAU<br><br>+ iCBT (3 times per week 30 min per visit, 69 modules presented on clinic computers)	IG:<br>N=92<br><br>CG:	Longest continuous abstinence, total abstinence, days retained in treatment	IG recipients exhibited on average 9.7 total days more of abstinence, 95% CI 2.3-17.2, reduced hazard of dropping out of treatment,

	criteria for buprenorphine treatment		CG: 12 weeks TAU (contingency management + biweekly 30 min f2f + buprenorphine dosing)	N=78		Hazard Ratio (HR)=0.47; 95% CI 0.26-0.85. Prior treatment for opioid dependence significantly moderated the additional improvement of IG for longest continuous days of abstinence
Ebert et al (2013), Germany<br><br>[59,83,84]	Inpatient treatment for mental disorder according to International Classification of Diseases-10th revision (ICD-10)	2-arm RCT	IG: 12 weeks TAU + iCBT (transdiagnostic Internet-based maintenance treatment [TIMT], with self-management module, asynchronous patient-coach communication, Internet patient support group) after inpatient treatment<br><br>CG: Access to TAU	IG:<br>N=200<br><br>CG:<br>N=200	General psychopathological symptom severity	mean $d_{between}$=0.48, effectiveness more pronounced among participants with a low (vs high) education level, participants with high (vs low) positive outcome expectations, and participants with anxiety disorder (vs mood disorder)
Golkaramnay et al (2007), Germany<br><br>[20]	Inpatient treatment for mental disorder according to ICD-10	Pre-post + control	IG: 12-15 weeks Internet group chat with a therapist for 90 min in open groups of 8-10 participants after discharge of inpatient treatment<br><br>CG: Access to TAU	IG:<br>N=114<br><br>CG:<br>N=114	Outcome Questionnaire-45 (OQ-45), Symptom Checklist-90-R (SCL-90-R), Giessener Beschwerde-bogen (GBB, Giessen list of complaints), Fragebogen zur Erfassung der Lebens-zufriedenheit (FLZ, Life Satisfaction Scale)	OQ-45: $d_{between}$=0.32[b]<br><br>SCL-90-R: $d_{between}$=0.27[a]<br><br>GBB: $d_{between}$=0.32[a]<br><br>12 months' follow-up: significantly more controls (38.5%, 40/104) negative outcome than participants (24.7%, 24/97)
Haug et al (2015),	Primary diagnosis of	2-arm RCT	IG: Stepped care:	IG:	CSR, BSQ (Body Sensation	Significantly better

Norway<br><br>[55]	panic disorder or social anxiety disorder according to DSM-IV, Clinician Severity Rating (CSR) ≥4		1. psychoeducation<br><br>2. iCBT<br><br>3. f2f-CBT<br><br>CG: Immediate f2f-CBT	N=85<br><br>CG:<br><br>N=88	Questionnaire), SPS, SIAS, SR-Composite	outcome of<br><br>CG on the outcome on CSR, no significant difference on the outcome on the SR-Composite
Høifødt et al (2013, 2015), Norway<br><br>[38,85]	Beck Depression Inventory-II (BDI-II) ≥10<41	2-arm RCT	IG: 7 weeks iCBT with 15-30 min f2f support after each module<br><br>CG: Access to TAU	IG:<br><br>N=52<br><br>CG:<br><br>N=54	BDI-II	$d_{between}$=0.65[b]<br><br>positive effect on probability of response: history of more depressive episodes, being married or cohabiting, scoring higher on a measure of life satisfaction<br><br>negative effect on probability of response: higher levels of dysfunctional thinking
Jacmon et al (2009), Australia<br><br>[46]	Major depression according to DSM-IV, BDI-II ≥14< 29	Pre-post	4-6 weeks Internet course on CBT skills with weekly email support, arrangement of individual f2f sessions as needed; average 3.7 f2f sessions	N=9	BDI-II, Hamilton Rating Scale for Depression (HDRS)	Large and significant pre-post reduction in BDI and HDRS, maintenance of improvement through 3-month follow-up
Kay-Lambkin et al (2011), Australia<br><br>[39]	BDI-II ≥17 + current problematic use of alcohol or cannabis	3-arm RCT	IG: Blended: 1 initial f2f session + 9 sessions iCBT with therapist support<br><br>CG1: f2f: 10 sessions f2f CBT	IG:<br><br>N=32<br><br>CG1:<br><br>N=35<br><br>CG2:	Agnew-Davies Relationship Measure (ARM), treatment attendance	Attendance rates equal between IG and CG1, 51% (34/67) completing all 10 treatment sessions;<br><br>no significant differences between IG and CG1 in ARM; subscale client initiative significantly higher in CG1 and

			CG2: brief intervention (BI), 1 individual f2f session	N=30		CG2 after session 5, this domain was related to better alcohol outcomes
Kenter, Warmerdam et al (2013), Netherlands<br><br>[50]	Symptoms of depression (BDI-II≥14) anxiety<br><br>(HADS >8) and/or work-related stress (Maslach Stress-Burnout Inventory [MBI] ≥2.2 on high emotional exhaustion + ≤2.2 depersonalization or ≤3.66 personal accomplishment)	Pre-post + control	IG: 5 weeks Internet problem-solving treatment with email feedback; subsequently f2f psychotherapy<br><br>CG: 5 weeks waiting list, then f2f psychotherapy	IG:<br><br>N=55<br><br>CG:<br><br>N=49	BDI-II, HADS-A, MBI	After 5 weeks:<br><br>depression $d_{between}$=0.94[b], anxiety $d_{between}$=1.07[b],<br><br>burnout $d_{between}$=−.07ns<br><br>after 12 weeks only significant difference for anxiety ($d_{between}$=0.69[b])
Kenter et al (2013, 2016), Kolovos et al (2016), Netherlands<br><br>[49,64,68]	DSM-IV diagnosis of major depressive disorder (MDD) assessed by Clinical International Diagnostic Interview (CIDI)	2-arm RCT	IG: 5 weeks Internet problem-solving treatment with email feedback; subsequently f2f psychotherapy<br><br>CG: 5 weeks waiting list + self-help problem-solving booklet, then subsequently f2f psychotherapy	IG:<br><br>N=136<br><br>CG:<br><br>N=133	CES-D,<br><br>cost effectiveness	IG: d_{within}=0.75[b]<br><br>CG: d_{within}=0.69[b]<br><br>$d_{between}$=0.07 ns<br><br>Mean societal costs for IG € 1579 higher than CG, ns
Kenwright et al (2001), United Kingdom<br><br>[21]	Anxiety symptoms, 20-min screening if anxiety problems were suitable for computer-guided treatment	Pre-post + control	IG: 4 sessions of 40-min iCBT (FearFighter) with 20-min f2f support<br><br>CG: 8 sessions f2f CBT	IG:<br><br>N=54<br><br>CG:<br><br>N=31	Fear Questionnaire (FQ), Work and Social Adjustment (WSA); main phobic trigger, main goal	Both groups improved similarly and highly significantly on most measures, IG spent 86% less time with a clinician than the CG
Kiluk et al (2016), United States	DSM-IV criteria for alcohol abuse or dependence	3-arm RCT	IG: 8 weeks TAU + biweekly iCBT (computer-based	IG:	Percent days abstinent (PDA)	d=0.71[a]

[35]			training for CBT, CBT4CBT, 7 modules of 45 min)<br><br>CG1: 8 weeks TAU (weekly individual or group psychotherapy sessions)<br><br>CG2: 8 weeks on-site iCBT (CBT4CBT) with brief weekly f2f monitoring	N=22<br><br>CG1:<br>N=22<br><br>CG2:<br>N=24		for the full sample<br><br>IG > CG1<br>$t_{536.4}$=2.68[a]
Klein et al (2012), United States<br><br>[60]	Patients discharged from residential drug and alcohol treatment, substance dependence according to DSM-IV	Empirical correlational study	Internet-based disease management program 18 months following discharge from treatment, including 7 modules iCBT, opportunities for fellowship with other recovering individuals, therapist support over email and telephone	N=1124	Number of modules accessed, continuous abstinence (binary), PDA from alcohol (continuous)	Significant relationship between number of modules accessed and substance outcomes in the year following treatment when controlling for motivation, self-efficacy, and pretreatment substance abuse
Kok et al (2014), Netherlands<br><br>[53]	DSM-IV-TR (Text Revision) or ICD-10 diagnosis of any phobia assessed by CIDI, wait-list for f2f psychotherapy	2-arm RCT	IG: 5 weeks iCBT exposure therapy with weekly student support; subsequently f2f psychotherapy<br><br>CG: 5 weeks waiting list + self-help exposure book, subsequently f2f psychotherapy	IG:<br>N=105<br><br>CG:<br>N=107	FQ	$d_{between}$=0.35[a]<br>5 weeks after baseline
Kooistra et al (2016), Netherlands<br><br>[26]	DSM-IV diagnosis of current depressive episode	Preliminary evaluation study	10 weeks 10 sessions f2f (45 min) + 10 sessions iCBT	N=9	System Usability Scale (SUS), CSQ-8	SUS: System usability above average (range 63-85), CSQ: mostly to very satisfied (range 16-32)

						Therapists: Evaluated blended treatment as helpful tool in providing evidence-based treatment
Kordy et al (2016), Germany<br><br>[37]	Recurrent MDD according to the Structured Clinical Interview for DSM-I (SCID-I) + history of at least three depressive episodes	3-arm RCT	IG1: 12-month TAU + iCBT (SUMMIT, supportive monitoring and depression management over the Internet)<br><br>IG2: 12-month TAU + iCBT (SUMMIT-PERSON: SUMMIT + clinician-guided individual + group chats)<br><br>CG: TAU including maintenance antidepressant medication and clinical management + individual crisis management plan (CMP) + project website	IG1:<br>N=77<br><br>IG2:<br>N=79<br><br>CG:<br>N=80	“Well” and “unwell” weeks over 24 months as determined by the Psychiatric Status Rating (PSR) of the Longitudinal Interval Follow-Up Evaluation (LIFE)	IG1: SUMMIT reduced unwell weeks compared with CG (Odds ratio [OR] 0.48; 95% CI 0.23-0.98) through faster transitions from unwell to well (OR 1.44; 95% CI 0.83-2.50) + slower transitions from well to unwell (OR 0.69; 95% CI 0.44-1.09), efficacy of SUMMIT strongest 8 months after the intervention<br><br>IG2: SUMMIT-PERSON not superior to either SUMMIT (OR 0.77; 95% CI 0.38-1.56) or TAU (OR 0.62; 95% CI 0.31-1.24)
Månsson et al (2013), Sweden<br><br>[27]	The self-rating scale of the Montgomery-Åsberg Depression Rating Scale (MADRS-S)<br><br>>30	Pre-post	Blended f2f and iCBT, 8-9 weeks	N=15	Beck Anxiety Inventory (BAI), Generalized Anxiety Disorder-7 (GAD-7), Patient Health Questionnaire-9 (PHQ-9), MADRS-S, and Quality of Life Inventory	Effect sizes from d_{within}=1.62 (PHQ-9) to 2.43 (MADRS-S), results maintained at the 12-month follow-up

					(QOLI)	
Marks et al (2004), United Kingdom [22]	DSM-IV diagnosis phobia or panic disorder + FQ Global Phobia Scale ≥4	3-arm RCT	IG: Blended: 6-hour-long iCBT sessions over 10 weeks, f2f support: up to 5 min at beginning, up to 15 min at the end<br>CG1: f2f: 6-hour-long individual treatment sessions over 10 weeks f2f CBT<br>CG2: Blended attention control: 6-hour-long sessions over 10 weeks guidance in self-relaxation techniques by a computer, f2f support: up to 5 min at beginning, up to 15 min at the end	IG: N=37<br>CG1: N=39<br>CG2: N=17	Self- and blind-assessor ratings of main problem and goals, FQ	Significant pre-post improvement for IG and CG1, significant between-group effects between IG and CG2 and between CG1 and CG2, no significant between-group effect between IG and CG1.<br>IG + CG2 73% less clinician time than entirely f2f group
Marsch et al (2016), Kim et al (2016), United States [86]	DSM-IV criteria for opioid dependence, be within first 30 days of methadone maintenance treatment program entry	2-arm RCT	IG: 52 weeks outpatient TAU + iCBT (TES, 62 interactive multimedia modules), iCBT substituting for about 30 min of standard care per week, 2 modules of about 15 min per session<br>CG: 52 weeks TAU: daily methadone maintenance doses, weekly or biweekly individual counseling sessions of up to 60 min	IG: N=80<br>CG: N=80	Urine toxicology results for opioid and cocaine abstinence for 52 weeks	IG significantly better opioid abstinence than CG, even among patients with a history of multiple addiction treatment episodes
Nordmo et al (2015),	Primary diagnosis Social	2-arm RCT	IG: 90-min session f2f + nine iCBT modules,	IG:	SPS, SIAS	Posttreatment d_{within}=0.70-0.95[a]

Norway<br><br>[44]	Anxiety Disorder assessed by<br><br>Mini-International Neuropsychiatric Interview (MINI) for at least 1 month, CSR ≥3		weekly 10-min therapist support by phone<br><br>CG: Nine iCBT modules, weekly 10-min therapist support by phone, no f2f	N=17<br><br>CG:<br><br>N=20		6-month follow-up d_{within}=0.70-1.00[a]<br><br>No significant differences between treatment conditions
Pier et al (2008), Shandley et al (2008), Australia<br><br>[40,87]	Primary diagnosis Panic Disorder assessed by<br><br>Anxiety Disorders Interview Schedule for DSM-IV (ADIS-IV)	Pre-post + control	IG: 12 weeks iCBT (Panic Online, PO) with f2f support by GP every 1-2 weeks<br><br>CG: 12 weeks iCBT (PO) only (email support every 1-2 weeks)	IG:<br><br>N=53<br><br>CG:<br><br>N=43	ADIS-IV, Anxiety Sensitivity Profile (ASP), Depression, Anxiety, and Stress Scale (DASS), Panic Disorder Severity Scale (PDSS), Treatment Credibility Scale-Modified (TCS-M), World Health Organization Quality of Life-BREF (WHOQOL-BREF),<br><br>attrition rate	Significant pre-post improvements for both groups in panic attack frequency, depression, anxiety, stress, anxiety sensitivity, agoraphobia avoidance, and quality of life maintained at follow-up; CG significantly higher physical ($F_{1,82}$=9.13[b]) and environmental ($F_{1,82}$=4.41[a]) quality of life and significantly lower attrition rate than IG
Robertson et al (2006), Australia<br>[36]	Current treatment for depression, diagnosis of major depressive episode	Pre-post	12 iCBT sessions (RecoveryRoad) with Internet progress monitoring over approximately 12 months as adjunct to usual treatment, clinicians had Internet access to patients' progress monitoring outcomes	N=144	iCBT adherence, self-reported medication adherence, DSS	Adherence 53% to 84%, depending on modality of reminder, self-reported medication adherence >90%. DSS: d_{within}=1.0

Sethi et al (2010), Australia<br><br>[45]	Low to moderate levels of depression or anxiety assessed by DASS	4-arm RCT	IG: Blended: five CBT sessions over 3 weeks, first half f2f CBT, second half iCBT (MoodGYM)<br><br>CG1: f2f: five CBT sessions over 3 weeks<br><br>CG2: iCBT: five MoodGYM sessions over 3 weeks<br><br>CG3: no intervention	IG:<br>N=9<br><br>CG1: N=10<br><br>CG2: N=10<br><br>CG3:<br>N=9	DASS-21, Kessler Psychological Distress Scale (K10), Automatic Thoughts Questionnaire-30 (ATQ-30)	IG > CG1 > CG2 > CG3<br><br>IG superior to CG1: anxiety (d=0.65^b)<br><br>and automatic negative thoughts (d=0.59^b)<br><br>IG superior to CG2:<br><br>depression, (d=0.87^b), anxiety (d=0.62^a), distress (d=0.64^a), and automatic negative thoughts (d=0.43^b)<br><br>CG2 superior to CG3: anxiety (d=0.54^a), distress d=0.81^a), and automatic negative thoughts (d=0.63^a).
Van Voorhees et al (2007), United States<br><br>[42]	Primary care patients (aged 18-24 years) at risk for depression (family history of clinical depression or personal history of depression)	Preliminary evaluation study	iCBT (11 modules) + 2 f2f sessions in primary care (one initial primary care motivational interview, one follow-up motivational interview)	N=14	Rating of fidelity, motivation, dose, perceived helpfulness, and potential costs of each component	Fidelity: 100% core concepts translated into intervention<br><br>Key motivations: risk reduction, intervention effectiveness, resiliency, altruism<br><br>Dose: 13 participants engaged iCBT, completing a mean of 7.2 modules (SD=3.9).<br><br>Perceived helpfulness: highest ratings at the 2 primary care interviews + the self-assessment and

						resiliency modules<br><br>Costs: duration of the 2 motivational interviews 17-18 min (similar to typical primary care visit)
Whitfield et al (2006), United Kingdom<br><br>[52]	Referrals on a clinical psychology waiting list, referral letter: depression or low mood as a major problem	Pre-post	Six hourly sessions iCBT, some f2f support from a self-help support nurse, one session per week	N=78	BDI-II, BAI, attendance rates	20 participants (26%) attended at least one session of iCBT, 14 completed all six sessions, BDI-II fell from a mean of 28.15 to 20.00 (*P*<.001), BAI fell from 20.30 to 14.55 (*P*=.021)
Wilhelmsen et al (2013), Norway<br><br>[47]	Primary care patients with mild to moderate depression	Qualitative study	Five iCBT modules, f2f support sessions of 20-30 min between modules	N=15	Semistructured interviews	Desired qualities by clients: acknowledgment, flexibility and feedback from a qualified therapist in the f2f consultations
Wright et al (2005), United States<br><br>[24]	Major depressive disorder according to SCID-I + interview with a clinical psychologist,<br><br>BDI ≥14	3-arm RCT	IG: 8 weeks of iCBT with f2f support; 9 sessions with 25-min f2f<br><br>CG1: 8 weeks f2f CBT, 9 sessions of 50 min<br><br>CG2: waiting list	IG:<br>N=15<br><br>CG1:<br>N=15<br><br>CG2:<br>N=15	HDRS, BDI	$d_{between}$ (CG1, CG2)=1.04[b],<br><br>$d_{between}$ (IG, CG2)=1.14[b],<br><br>$d_{between}$ (IG, CG1)=0.10, ns
Zwerenz et al (2015), Germany<br><br>[61]	Inpatient psychotherapy, ICD-10 disorder of depression	2-arm RCT	IG: Inpatient psychotherapy + 12-week Internet-based self-help treatment (deprexis), 12 modules, beginning during inpatient treatment	IG:<br>N=120<br><br>CG:<br>N=120	BDI	Study protocol

			CG: Inpatient psychotherapy + 12 weeks access to an Internet platform with weekly updated information on depression			

[a]Significant at the .05 level.

[b]Significant at the .01 level.
