# Supplementary material for: Blending Face-to-Face and Internet-Based Interventions for the Treatment of Mental Disorders in Adults: Systematic Review
Source: J Med Internet Res. 2017 Sep 15;19(9):e306. doi: 10.2196/jmir.6588 (PMC5622288; doi:10.2196/jmir.6588)
Supplement: Multimedia Appendix 3 [file jmir_v19i9e306_app3.pdf]

Study quality assessment of blended treatments.

Study (year), country	Study design	Randomization	Report of statistics	Sample size	Nonblended control group	Sum
Anderson et al (2016), Sweden [43]	2	2	2	0	0	6
Braamse et al (2010, 2016), Netherlands [54,74]	2	2	2	0	1	7
Brakemeier et al (2013), Germany [58]	0	0	0	0	0	0
Callan et al (2009), United States [19]	0	0	0	0	0	0
Campbell et al (2012, 2014, 2015), Cochran et al (2015), Cunningham et al (2015), Murphy et al (2016), Tofighi et al (2016), United States [29,31,56,69,75-77]	2	2	1	2	2	9
Campbell et al (2015), United States [41]	1	0	1	0	0	2
Carroll et al (2008, 2009, 2011), Sugarman et al (2010), Olmstead et al (2010), United States [63,65,78-80]	2	2	2	0	1	7
Carroll et all (2014, 2015), Morie et al (2015), United States [34,81,82]	2	2	2	0	1	7
Christensen et al (2014), United States [32]	2	2	1	1	1	7

Ebert et al (2013), Germany<br><br>[59,83,84]	2	2	2	2	0	8
Golkaramnay et al (2007), Germany<br><br>[20]	2	0	2	1	0	5
Hantsoo et al (2014),<br><br>United States<br><br>[48]	0	0	0	0	0	0
Härter et al (2015), Germany<br><br>[57]	2	2	0	2	1	7
Haug et al (2015), Norway<br><br>[55]	2	2	1	1	1	7
Høifødt et al (2013, 2015), Norway<br><br>[38,85]	2	2	2	0	0	6
Jacmon et al (2019) Australia<br><br>[4]	1	0	1	0	0	2
Kay-Lambkin et al (2011), Australia<br><br>[39]	2	2	1	0	2	7
Kemmeren et al (2016), Netherlands<br><br>[25,64]	2	2	0	1	1	6
Kenter, Warmerdam et al (2013), Netherlands<br><br>[50]	2	0	2	0	0	4
Kenter et al (2013, 2016), Kolovos et al (2016) Netherlands<br><br>[49,64,68)	2	2	2	2	2	10
Kenwright et al (2001), United Kingdom<br><br>[21]	2	0	1	0	2	5

Kiluk et al (2016), United States<br><br>[35]	2	2	2	0	1	7
Klein et al (2012), United States<br><br>[60]	1	0	1	2	0	4
Kok et al (2014), Netherlands<br><br>[53]	2	2	2	1	2	9
Kooistra et al (2014), Netherlands<br><br>[15]	2	2	0	1	2	7
Kooistra et al (2016), Netherlands<br><br>[26]	1	0	1	0	0	2
Kordy et al (2016), Germany<br><br>[37]	2	2	1	1	1	7
Krieger et al (2014), Germany<br><br>[28]	2	2	0	2	1	7
Månsson et al (2013), Sweden<br><br>[27]	1	0	2	0	0	3
Marks et al (2014), United Kingdom<br><br>[22]	2	2	2	0	2	8
Marsch et al (2016), Kim et al (2016), United States<br><br>[86]	2	2	1	1	2	8
Nordmo et al (2015), Norway<br><br>[44]	2	2	2	0	1	7
Pier et al (2008), Shandley et al (2008), Australia<br><br>[40,87]	2	0	1	0	2	5
Reins et al (2013), Germany<br><br>[51]	2	2	0	1	0	5
Robertson et al (2006), Australia<br>[36]	1	0	2	1	0	4

Romijn et al (2015), Netherlands<br><br>[23]	2	2	0	1	2	7
Sethi et al (2010), Australia<br><br>[45]	2	2	2	0	2	8
Tannenbaum and Spiranovic (2010), Australia<br><br>[62]	0	0	0	0	0	0
Van Straten et al (2010), Netherlands<br><br>[56]	2	2	0	1	1	6
Van Voorhees et al (2007), United States<br><br>[42]	1	0	1	0	0	2
Whitfield et al (2006), United Kingdom<br><br>[52]	1	0	1	0	0	2
Wilhelmsen et al (2013), Norway<br><br>[47]	1	0	0	0	0	1
Wright et al (2005), United States<br><br>[24]	2	2	2	0	2	8
Zwerenz et al (2015), Germany<br><br>[61]	2	2	0	1	1	6
